# Supplementary material for: Influence of N6-methyladenosine (m6A) modification on cell phenotype in Alzheimer’s disease
Source: PLoS One. 2023 Aug 7;18(8):e0289068. doi: 10.1371/journal.pone.0289068 (PMC10406241; doi:10.1371/journal.pone.0289068)
Supplement: Attachment — Submitted filename: Comments.docx [file pone.0289068.s001.docx]

Dear Editor,

The manuscript titled “Influence of N6-Methyladenosine (m6A) Modification on Cell Phenotype in Alzheimer’s Disease’’ tries to understand the role of methyl adenosine in Alzheimer’s disease using insilico biology. “This manuscript needs the following major revision before being accepted”. Also please be informed that, I have limited knowledge in bioinformatics databases and the methodologies used needs a validation from bioinformatician.

Comments

1. Overall English could be improved
2. we found 1277 differently expressed genes in brain tissues of AD patients compared with normal brain tissues. Of these, 498 were up-regulated and 779 were down-regulated. A recent article using the GSE5281 2,063 DEGs comprising 854 upregulated genes and 1,209 downregulated genes were obtained based on microarray analysis of GSE5281 ( <https://www.karger.com/Article/Abstract/518727>) . Why there exists discrepancy in DEG’s
3. Can the cross -genes could be renamed as overlapping genes as the name seems to be confusing.
4. Using the venn diagram method the authors integrated differential expression data from GSE5281 and GeneCards database. Whether the presence of m6A modifications were validated in the overlapping genes which formed the basis for their model.
5. It would be interesting to show m6A modifications using sequencing data atleast for two overlapping genes atleast supporting data
