# Supplementary material for: Influence of N6-methyladenosine (m6A) modification on cell phenotype in Alzheimer’s disease
Source: PLoS One. 2023 Aug 7;18(8):e0289068. doi: 10.1371/journal.pone.0289068 (PMC10406241; doi:10.1371/journal.pone.0289068)
Supplement: Attachment — Submitted filename: Response to Reviewers.docx [file pone.0289068.s002.docx]

Dear reviewers,

We thank you very much for giving us an opportunity to revise our manuscript. We appreciate editor and reviewers very much for your positive and constructive comments and suggestions on our manuscript. The comments and suggestions are all valuable and very helpful for revising and improving our paper, as well as the important guiding significance to our researches. We have studied comments carefully and have made correction which we hope meet with approval.

In this revised version, we have addressed the concerns of the reviewers. An point-by-point response to the reviewers' comments is enclosed. We hope that these revisions successfully address their concerns and requirements and that this manuscript will be accepted.

Best wishes,

Pengyun Ni

**Reviewer #1:**

In addition, the authors must further clearly describe their crucial findings on biological functions that benefits the field in abstract and other relevant sections clearly.

**Response：**As you suggested, we have described biological functions and signaling pathways in the abstract, conclusions, and discussion sections.

Major Comments

• Introduction section should be expanded in few sentences stating about how mechanistically m6A modification occurs in mammals. Also, a sentence about demethylation.

**Response：**As you suggested, we have added in the introduction a key mechanism by which M6A modification occurs in mammals.

•In fig. 1 and 2, the authors showed DEGs and m6A associated genes. However, the authors should also show the differences in genes associated with hyper and hypo methylation.

**Response：**As you suggested, we have identified some of the cross-expressed overlopping genes for DEGs and m6A in Fig 1. Red colour indicates up-regulated genes, and blue indicates down-regulated genes. In addition, we show the expression of up-regulated and down-regulated genes between AD and control groups by PCA. PCA shows that the Control group and AD group samples were separated, indicating that there were differences in gene expression of up-regulated and down-regulated overlapping genes different between the groups.

• Have the authors attempted to study DEGs and GO (figs 1-5) in the brain tissue specific manner? It would be interesting to study tissue specific differences and the influences of m6A modifications.

**Response：**We took the resulting 10 hub genes expression levels into the GSE5281 and GSE48350 datasets, respectively, and compared the hub genes expression levels between the AD and control groups. The value of hub gene expression and ROC in the diagnosis of AD was verified. At the same time, we find the expression level and score of hub genes in BGEE database to validate the expression of hub genes in AD.

Exceptly, I’m so sorry that our team does not have the equipment and reagents to do translational research. Because our hospital belong to the prefecture-level City Hospital of traditional Chinese medicine which is based on clinical work and clinical research. In addition, this research has not been supported by any funds. It does not have the enough fund for further experimental validation study. If there are sufficient conditions to support our further supplement experimental study, we would validate the mechanisms of action and the signaling pathways of molecules and protein targets in the findings of this study.

• Why the authors used only one dataset, GSE5281 in their study. The authors should include multiple available datasets which have different sources of tissue samples for a better interpretation.

**Response：**Our initial idea for this study was to study human specimens, as this is more in line with the actual state of the patient. Therefore, in the selection of the data set, the species is restricted to homo. Through your suggestion, We realized the imprecision of using only one data set. We then consulted an experienced teacher who suggested that it would be more scientific to select a data set from the same platform. In addition, in order to ensure sufficient sample size and valid data, we select data sets with sample size greater than 50.

So our criteria for selecting the dataset were: the disease name was“Alzheimer's disease”, the species was “homo sapiens”, GPL570 (the same platform as GSE5281) , and valid data were available for GEO2R analysis. Finally, we selected GSE5281 and GSE48350 as the data sources in the GPL570 platform.

• Have the authors attempted to check statistical significance in fig 7?

**Response：**The data in Fig 7 are derived from the HPA (Human Protein Atlas) database and the BGEE database. The data in these two databases were obtained by counting the corresponding human tissue specimens. In the database, we can only see the average value of gene expression in tissues, and the standard deviation or error value can not be collected, so we did not do statistical significance analysis. Only by describing and analyzing the average of gene expression, we can verify the expression of the core genes in each brain region.

But we took 10 hub genes into GSE5821 and GSE48350 to statistically analyze genes expression levels, and we did a statistical analysis of gene expression differences between AD and normal controls. You will see it on the figures 8A-8D in the manuscript.

Minor Comments

• Indicate the threshold cut-off used for PPI analysis.

**Response：**As you suggested, we added a threshold cut-of for PPI.

• The authors should make a table for top 10 genes from KEGG. The authors should revisit fig. 3 legend as I don’t see dot plot in fig. 3.

**Response：**As you suggested, we have supplemented the tables for the results of the GO and KEGG analyses and modified the legend for Fig. 3.

**Reviewer #2:**

1.Overall English could be improved.

**Response：**We asked experienced teacher to polish our paper and carefully checked the spelling, grammar and formatting. And we also asked professional editors to review our articles for revisions. We believe the revised version will meet the English presentation standard.

2.We found 1277 differently expressed genes in brain tissues of AD patients compared with normal brain tissues. Of these, 498 were up-regulated and 779 were down-regulated. A recent article using the GSE5281 2,063 DEGs comprising 854 upregulated genes and 1,209 downregulated genes were obtained based on microarray analysis of GSE5281 ( https://www.karger.com/Article/Abstract/518727) . Why there exists discrepancy in DEG’s.

**Response：**We noticed in the materials and methods section of that article showed that"We identified DEGs by using the Limma package in ***R software (version 3.5.2) . Log2FC >1 and FDR ≤0.05***”. At the time, we used the GSE5281 data set to filter DEGs based on“***|log2(Fold Change)| > 1 and P.value < 0.05*** “ and used the Limma package in ***R software (version 4.2.1)*** .

On the one hand, two articles had different screening conditions for DEGs. On the other hand, the two articles use different versions of R software. In some GEO data analysis lectures, the teachers mentioned that different computers and R software screen DEGs because the background parameters of the computers and R software are not set in the same way, even with the same data set and the same filter conditions, the resulting DEGs will be different and can not be exactly the same.

3.Can the cross -genes could be renamed as overlapping genes as the name seems to be confusing.

**Response：**We have modified it according to your suggestion.

4.Using the venn diagram method the authors integrated differential expression data from GSE5281 and GeneCards database. Whether the presence of m6A modifications were validated in the overlapping genes which formed the basis for their model.

**Response：**We took the resulting 10 hub genes expression levels into the GSE5281 and GSE48350 datasets, respectively, and compared the hub genes expression levels between the AD and control groups. The value of hub gene expression and ROC in the diagnosis of AD was verified. At the same time, we find the expression level and score of hub genes in BGEE database to validate the expression of hub genes in AD.

Exceptly, I’m so sorry that our team does not have the equipment and reagents to do the verification experiment.. Because our hospital belong to the prefecture-level City Hospital of traditional Chinese medicine which is based on clinical work and clinical research. In addition, this research has not been supported by any funds. It does not have the enough fund for further experimental validation study. If there are sufficient conditions to support our further supplement experimental study, we would validate the mechanisms of action and the signaling pathways of molecules and protein targets in the findings of this study.

5.It would be interesting to show m6A modifications using sequencing data atleast for two overlapping genes atleast supporting data.

**Response：**As you suggest, we consider that it is more scientific to choose a data set on the same platform. In addition, in order to ensure sufficient sample size and valid data, we select data sets with sample size greater than 50. So our criteria for selecting the dataset were: the disease name was“Alzheimer's disease”, the species was “homo sapiens”, GPL570 (the same platform as GSE5281) , and valid data were available for GEO2R analysis. Finally, we selected GSE5281 and GSE48350 as the data sources in the GPL570 platform.
