# Supplementary material for: Influence of N6-methyladenosine (m6A) modification on cell phenotype in Alzheimer’s disease
Source: PLoS One. 2023 Aug 7;18(8):e0289068. doi: 10.1371/journal.pone.0289068 (PMC10406241; doi:10.1371/journal.pone.0289068)
Supplement: Attachment — Submitted filename: Response to Reviewers.docx [file pone.0289068.s003.docx]

Dear reviewers,

We are very grateful to the reviewers for your recognition of our research work and for giving us expectations for progress. We can also further improve this study under the guidance of the reviewers. Meantime, We also understand that the experimental verification of the conclusions of this study will be more convincing and scientific. However, the equipment, funding, and research resources currently possessed by our team cannot support our requirements for experimental verification. In our plan, if this manuscript is published, we may be able to apply for some research funding or cooperation opportunities. Thus, a series of animal experiments and cell experiments can be carried out. For example, we may be able to conduct research on the mechanism of Alzheimer's disease by upregulating or knocking out core genes in the Hippo signaling pathway, and verify the biological processes, cellular components, and molecular functions conclusions in this manuscript. I am sorry that we are currently unable to conduct relevant experimental research in our current situation. I hope to receive your understanding.

Best wishes,

Pengyun Ni
